# *rac*-2-[(2-Chloro­phen­yl)(4-chloro­phen­yl)meth­yl]-1,3-dioxolane

**DOI:** 10.1107/S1600536812023781

**Published:** 2012-06-13

**Authors:** Daniela F. Maluf, Sailer S. dos Santos, Claudia C. Gatto, Brás H. de Oliveira

**Affiliations:** a Departament of Pharmacy, Federal University of Paraná - UFPR, 81531-990, Curitiba - PR, Brazil; b Departament of Science and Technology, State University of Santa Cruz - UESC, 45662-900, Ilhéus - BA, Brazil; c Institute of Chemistry, University of Brasília - UnB, 70904-970, Brasília - DF, Brazil; d Departament of Chemistry, Federal University of Paraná - UFPR, 81531-990, Curitiba - PR, Brazil

## Abstract

The title compound, C_16_H_14_Cl_2_O_2_, is a chiral mitotane derivative that contains a dioxolane ring and crystallizes from methanol as a racemic mixture. It was obtained in high yield from mitotane and ethyl­eneglycol in alkaline medium, followed by neutralization with sulfuric acid and extraction with ethyl acetate. The mol­ecular structure is stabilized by an intra­molecular C— H⋯ O hydrogen bond. The dihedral angle between the aromatic rings is 80.1 (2)°. The dioxolane ring adopts a puckered envelope conformation with an O atom as the flap.

## Related literature

For related dioxolane geometry, see: Bolte *et al.* (1997 ▶). For organochlorines, see: Smith & Bennett (1977 ▶); Canti­llana & Eriksson (2009 ▶); Jabbar *et al.* (2006 ▶). For dechlorination of organochlorine compounds, see: Grummitt *et al.* (1946 ▶). For their adrenolytic activity, see: Fassnacht *et al.* (2010 ▶); Berruti *et al.* (2005 ▶). For organochlorine as insecticide metabolites in bioremediation studies, see: Purnomo *et al.* (2011 ▶); Fuentes *et al.* (2010 ▶); Matsumoto *et al.* (2009 ▶). For the use of mitotane [systematic name: 2-(2-chloro­phen­yl)-2-(4-chloro­phen­yl)-1,1-dichloro­ethane] in adrenocortical carcinoma treatment, see: Maluf *et al.* (2011 ▶); Rosati *et al.* (2008) ▶; Terzolo *et al.* (2007 ▶). For structure–activity studies of mitotane derivatives, see: Bleiberg & Larson (1973 ▶); Schteingart *et al.* (1993 ▶).
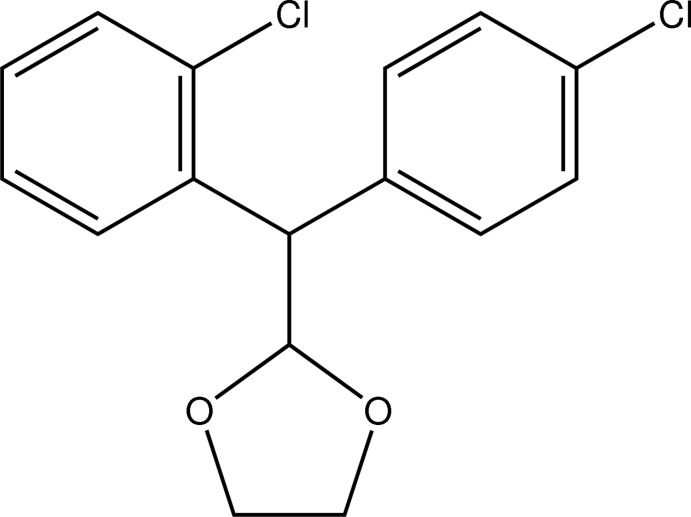


## Experimental

### 

#### Crystal data

C_16_H_14_Cl_2_O_2_
*M* *_r_* = 309.17
Triclinic, 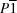

*a* = 7.5728 (2) Å
*b* = 10.2268 (2) Å
*c* = 11.2858 (2) Å
α = 63.357 (1)°
β = 84.021 (1)°
γ = 71.194 (1)°
*V* = 738.68 (3) Å^3^
*Z* = 2
Mo *K*α radiation
μ = 0.44 mm^−1^
*T* = 296 K
0.59 × 0.56 × 0.29 mm

#### Data collection

Bruker SMART APEXII CCD diffractometer
Absorption correction: multi-scan (*SADABS*; Bruker, 2009 ▶) *T*_min_ = 0.783, *T*_max_ = 0.883
24953 measured reflections
4556 independent reflections
3654 reflections with *I* > 2σ(*I*)
*R*_int_ = 0.022

#### Refinement

*R*[*F* ^2^ > 2σ(*F* ^2^)] = 0.040
*wR*(*F* ^2^) = 0.117
*S* = 1.05
4556 reflections
181 parameters
H-atom parameters constrained
Δρ_max_ = 0.37 e Å^−3^
Δρ_min_ = −0.29 e Å^−3^

### 

Data collection: *APEX2* (Bruker, 2009 ▶); cell refinement: *SAINT* (Bruker, 2009 ▶); data reduction: *SAINT*; program(s) used to solve structure: *SHELXTL* (Sheldrick, 2008 ▶); program(s) used to refine structure: *SHELXTL*; molecular graphics: *ORTEP-3 for Windows* (Farrugia, 1997 ▶); software used to prepare material for publication: *SHELXTL*.

## Supplementary Material

Crystal structure: contains datablock(s) I, global. DOI: 10.1107/S1600536812023781/bx2412sup1.cif

Structure factors: contains datablock(s) I. DOI: 10.1107/S1600536812023781/bx2412Isup2.hkl

Supplementary material file. DOI: 10.1107/S1600536812023781/bx2412Isup3.cml

Additional supplementary materials:  crystallographic information; 3D view; checkCIF report

## Figures and Tables

**Table 1:** Hydrogen-bond geometry (Å, °)

*D*—H⋯*A*	*D*—H	H⋯*A*	*D*⋯*A*	*D*—H⋯*A*
C12—H12⋯O1	0.93	2.38	3.046 (2)	128

**Fig. 1.**
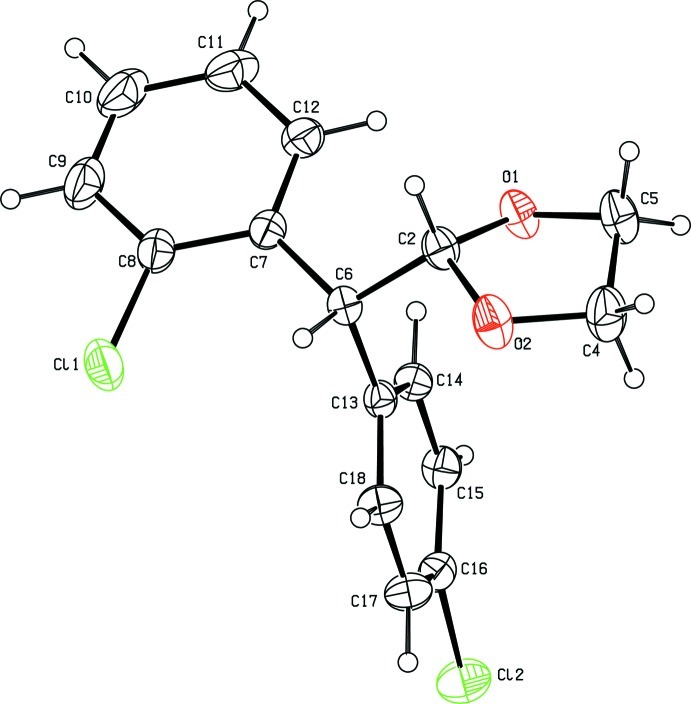
The molecular structure of the title compound, with atom labels and 30% probability displacement ellipsoids for non-H atoms.

## supplementary crystallographic information

### Comment

The title compound, which crystallizes from methanol as a racemic mixture, has been obtained after C_1_ oxidation and dechlorination of 2-(2-chlorophenyl)-2-(4-chlorophenyl)-1,1-dichloroethane, also known as mitotane or o,p'-DDD. The reaction generated an additional structural feature in the molecule, the dioxolane ring. While organochlorine compounds are widely described in the literature as insecticide metabolites in bioremediation studies (Purnomo *et al.*, 2011; Fuentes *et al.*, 2010; Matsumoto *et al.*, 2009), mitotane itself is a drug used exclusively for adrenocortical carcinoma treatment (Maluf *et al.*, 2011; Rosati *et al.*, 2008, Terzolo *et al.*, 2007). However, mitotane therapy produces important side effects due to its toxicity. Therefore, derivatives have been prepared in order to overcome those limitations. Several studies of structure-activity relationship report that the substitution of the hydrogen at the C_1_ position of mitotane results in the loss of activity and the use of the o,p'-DDD isomer – which refers to a specific substitution pattern in the aromatic rings – leads to a better pharmacological effect than that provided by the m,p' and p,p' isomers (Bleiberg and Larson, 1973; Schteingart *et al.*, 1993). Search for new compounds that keep the single hydrogen bound to C_1_ and also the o,p'-substitution in the aromatic rings is necessary for an improved treatment of this malignant cancer. The molecule described herein is a good example of a mitotane derivative that presents these structural features relevant for adrenolytic activity. The molecular structure of the title compound is depicted in Figure 1. Bond lenghts and angles are as expected. The dioxolane ring adopts a puckered envelope conformation with C_2_, O_2_, C_4_ and C_5_ in the same plane, with the O_1_ atom placed about 0.4661 (1) Å above it. The coplanar atoms of the dioxolane ring form a dihedral angle of 74.63 (3)° with *p*-chloro-phenyl ring and an angle of 9.83 (3)° with the *o*-chloro-phenyl ring. The angle between the aromatic groups is 80.1 (2)°. The molecular structure is stabilized by an intramolecular C— H··· O hydrogen bond interaction (C···O 3.046 (2)Å; C—H···O 128° ). Weak C—H···Cl is also observed.

### Experimental

Mitotane (o,p'-DDD) was added to a mixture of ethylene glycol, KOH and water. The reaction was carried out overnight under reflux at 137°C. After this period, the reaction mixture was cooled down to room temperature and diluted with water. Concentrated sulfuric acid (98%) was then added to take the solution pH down to 3.0. The salt formed was removed by filtration on a Büchner funnel. The filtrate was extracted with ethyl acetate, the organic layer was concentrated by rotary evaporation and the oily yellow residue was redissolved in warm methanol (30°C). Thin, colorless plate-like crystals suitable for X-ray diffraction analysis were obtained from this methanol solution. Total reaction yield: 84%.

### Refinement

All H-atoms were positioned geometrically and refined using a riding model, with C—H = 0.93—0.98 Å and *U*_iso_(H) =1.2*U*_eq_(C).

### Crystal data

**Table:**

C_16_H_14_Cl_2_O_2_	*Z* = 2
*M**_r_* = 309.17	*F*(000) = 320
Triclinic, *P*1	*D*_x_ = 1.39 Mg m^−^^3^
*a* = 7.5728 (2) Å	Mo *K*α radiation, λ = 0.71073 Å
*b* = 10.2268 (2) Å	Cell parameters from 4556 reflections
*c* = 11.2858 (2) Å	θ = 4.2–57.4°
α = 63.357 (1)°	µ = 0.44 mm^−^^1^
β = 84.021 (1)°	*T* = 296 K
γ = 71.194 (1)°	Block, colourless
*V* = 738.68 (3) Å^3^	0.59 × 0.56 × 0.29 mm

### Data collection

**Table:**

Bruker SMART APEXII CCD diffractometer	4556 independent reflections
Radiation source: sealed tube	3654 reflections with *I* > 2σ(*I*)
Graphite monochromator	*R*_int_ = 0.022
phi & ω scans	θ_max_ = 30.7°, θ_min_ = 2.0°
Absorption correction: multi-scan (*SADABS*; Bruker, 2009)	*h* = −10→10
*T*_min_ = 0.783, *T*_max_ = 0.883	*k* = −14→14
24953 measured reflections	*l* = −16→16

### Refinement

**Table:**

Refinement on *F*^2^	Primary atom site location: structure-invariant direct methods
Least-squares matrix: full	Secondary atom site location: difference Fourier map
*R*[*F*^2^ > 2σ(*F*^2^)] = 0.040	H-atom parameters constrained
*wR*(*F*^2^) = 0.117	*w* = 1/[σ^2^(*F*_o_^2^) + (0.0556*P*)^2^ + 0.1712*P*] where *P* = (*F*_o_^2^ + 2*F*_c_^2^)/3
*S* = 1.05	(Δ/σ)_max_ = 0.001
4556 reflections	Δρ_max_ = 0.37 e Å^−^^3^
181 parameters	Δρ_min_ = −0.29 e Å^−^^3^
0 restraints	

### Special details

**Table:**

Geometry. All s.u.'s (except the s.u. in the dihedral angle between two l.s. planes) are estimated using the full covariance matrix. The cell s.u.'s are taken into account individually in the estimation of s.u.'s in distances, angles and torsion angles; correlations between s.u.'s in cell parameters are only used when they are defined by crystal symmetry. An approximate (isotropic) treatment of cell s.u.'s is used for estimating s.u.'s involving l.s. planes.
Refinement. Refinement of *F*^2^ against ALL reflections. The weighted *R*-factor *wR* and goodness of fit *S* are based on *F*^2^, conventional *R*-factors *R* are based on *F*, with *F* set to zero for negative *F*^2^. The threshold expression of *F*^2^ > 2σ(*F*^2^) is used only for calculating *R*-factors(gt) *etc*. and is not relevant to the choice of reflections for refinement. *R*-factors based on *F*^2^ are statistically about twice as large as those based on *F*, and *R*- factors based on ALL data will be even larger.

### Fractional atomic coordinates and isotropic or equivalent isotropic displacement parameters (Å^2^)

**Table:**

	*x*	*y*	*z*	*U*_iso_*/*U*_eq_	
Cl1	0.77214 (6)	0.38255 (5)	0.01175 (4)	0.06217 (13)	
Cl2	1.10734 (6)	−0.41352 (5)	0.31682 (5)	0.06334 (13)	
C6	0.51390 (16)	0.19844 (14)	0.17862 (11)	0.0346 (2)	
H6	0.5131	0.2544	0.0818	0.041*	
C13	0.65594 (16)	0.04056 (14)	0.21569 (11)	0.0335 (2)	
C15	0.86840 (19)	−0.19344 (15)	0.37708 (13)	0.0417 (3)	
H15	0.9191	−0.2545	0.4634	0.05*	
C14	0.73344 (18)	−0.05219 (15)	0.34448 (12)	0.0379 (2)	
H14	0.6938	−0.0186	0.4098	0.046*	
C7	0.57303 (16)	0.28987 (14)	0.23302 (13)	0.0377 (2)	
C16	0.92668 (18)	−0.24239 (15)	0.27971 (14)	0.0414 (3)	
C2	0.31576 (17)	0.19008 (16)	0.21133 (13)	0.0406 (3)	
H2	0.2303	0.2932	0.1918	0.049*	
C17	0.8485 (2)	−0.15571 (17)	0.15211 (14)	0.0479 (3)	
H17	0.8857	−0.1914	0.088	0.058*	
C18	0.7135 (2)	−0.01446 (16)	0.12117 (13)	0.0437 (3)	
H18	0.6605	0.0446	0.0353	0.052*	
C8	0.69189 (19)	0.37655 (15)	0.16361 (16)	0.0458 (3)	
C9	0.7506 (2)	0.4593 (2)	0.2126 (2)	0.0655 (5)	
H9	0.8287	0.5168	0.1639	0.079*	
C5	0.1532 (2)	0.0332 (2)	0.34243 (16)	0.0560 (4)	
H5A	0.033	0.1052	0.3428	0.067*	
H5B	0.1653	−0.0658	0.4177	0.067*	
C12	0.5178 (2)	0.28940 (17)	0.35447 (16)	0.0483 (3)	
H12	0.4384	0.2333	0.4035	0.058*	
C11	0.5776 (3)	0.3703 (2)	0.4049 (2)	0.0630 (4)	
H11	0.5397	0.3666	0.4872	0.076*	
C4	0.1763 (3)	0.0197 (2)	0.21519 (17)	0.0620 (4)	
H4A	0.2555	−0.0822	0.2289	0.074*	
H4B	0.0563	0.0397	0.1765	0.074*	
C10	0.6927 (3)	0.4557 (2)	0.3333 (2)	0.0729 (5)	
H10	0.7314	0.511	0.3664	0.088*	
O1	0.30232 (14)	0.08905 (13)	0.34445 (9)	0.0480 (2)	
O2	0.26110 (15)	0.13305 (14)	0.13264 (10)	0.0536 (3)	

### Atomic displacement parameters (Å^2^)

**Table:**

	*U*^11^	*U*^22^	*U*^33^	*U*^12^	*U*^13^	*U*^23^
Cl1	0.0564 (2)	0.0582 (2)	0.0640 (2)	−0.02759 (18)	0.01797 (18)	−0.01678 (18)
Cl2	0.0595 (2)	0.0481 (2)	0.0721 (3)	0.00034 (16)	−0.00404 (19)	−0.02775 (19)
C6	0.0336 (5)	0.0369 (6)	0.0318 (5)	−0.0132 (4)	0.0005 (4)	−0.0123 (4)
C13	0.0329 (5)	0.0370 (6)	0.0337 (5)	−0.0155 (4)	0.0016 (4)	−0.0151 (4)
C15	0.0460 (7)	0.0399 (6)	0.0344 (6)	−0.0138 (5)	−0.0027 (5)	−0.0109 (5)
C14	0.0439 (6)	0.0405 (6)	0.0315 (5)	−0.0151 (5)	0.0027 (4)	−0.0165 (5)
C7	0.0319 (5)	0.0330 (5)	0.0463 (6)	−0.0083 (4)	−0.0025 (5)	−0.0161 (5)
C16	0.0401 (6)	0.0364 (6)	0.0484 (7)	−0.0119 (5)	0.0004 (5)	−0.0188 (5)
C2	0.0348 (6)	0.0478 (7)	0.0385 (6)	−0.0163 (5)	−0.0002 (4)	−0.0157 (5)
C17	0.0540 (8)	0.0516 (8)	0.0451 (7)	−0.0119 (6)	−0.0003 (6)	−0.0297 (6)
C18	0.0487 (7)	0.0488 (7)	0.0348 (6)	−0.0119 (6)	−0.0042 (5)	−0.0205 (5)
C8	0.0371 (6)	0.0369 (6)	0.0601 (8)	−0.0121 (5)	0.0005 (5)	−0.0177 (6)
C9	0.0532 (9)	0.0539 (9)	0.1019 (14)	−0.0269 (7)	0.0039 (9)	−0.0379 (9)
C5	0.0488 (8)	0.0723 (10)	0.0500 (8)	−0.0347 (7)	0.0054 (6)	−0.0195 (7)
C12	0.0481 (7)	0.0489 (7)	0.0559 (8)	−0.0162 (6)	0.0048 (6)	−0.0295 (7)
C11	0.0651 (10)	0.0654 (10)	0.0756 (11)	−0.0147 (8)	−0.0005 (8)	−0.0483 (9)
C4	0.0702 (10)	0.0756 (11)	0.0547 (9)	−0.0449 (9)	0.0033 (7)	−0.0260 (8)
C10	0.0665 (11)	0.0683 (11)	0.1120 (16)	−0.0252 (9)	−0.0017 (10)	−0.0592 (12)
O1	0.0450 (5)	0.0713 (7)	0.0346 (4)	−0.0322 (5)	0.0057 (4)	−0.0203 (4)
O2	0.0545 (6)	0.0796 (7)	0.0372 (5)	−0.0400 (6)	−0.0004 (4)	−0.0209 (5)

### Geometric parameters (Å, º)

**Table:**

Cl1—C8	1.7388 (16)	C17—C18	1.387 (2)
Cl2—C16	1.7402 (13)	C17—H17	0.93
C6—C7	1.5146 (17)	C18—H18	0.93
C6—C13	1.5189 (16)	C8—C9	1.390 (2)
C6—C2	1.5278 (17)	C9—C10	1.375 (3)
C6—H6	0.98	C9—H9	0.93
C13—C18	1.3885 (18)	C5—O1	1.4261 (17)
C13—C14	1.3926 (16)	C5—C4	1.491 (2)
C15—C16	1.3794 (19)	C5—H5A	0.97
C15—C14	1.3843 (19)	C5—H5B	0.97
C15—H15	0.93	C12—C11	1.390 (2)
C14—H14	0.93	C12—H12	0.93
C7—C12	1.389 (2)	C11—C10	1.373 (3)
C7—C8	1.3980 (18)	C11—H11	0.93
C16—C17	1.380 (2)	C4—O2	1.4181 (19)
C2—O1	1.4067 (16)	C4—H4A	0.97
C2—O2	1.4131 (17)	C4—H4B	0.97
C2—H2	0.98	C10—H10	0.93
			
C7—C6—C13	111.98 (9)	C17—C18—H18	119.3
C7—C6—C2	113.89 (10)	C13—C18—H18	119.3
C13—C6—C2	112.22 (10)	C9—C8—C7	121.93 (15)
C7—C6—H6	106	C9—C8—Cl1	117.88 (12)
C13—C6—H6	106	C7—C8—Cl1	120.19 (11)
C2—C6—H6	106	C10—C9—C8	119.73 (16)
C18—C13—C14	118.17 (12)	C10—C9—H9	120.1
C18—C13—C6	120.66 (11)	C8—C9—H9	120.1
C14—C13—C6	121.16 (11)	O1—C5—C4	102.77 (12)
C16—C15—C14	119.10 (12)	O1—C5—H5A	111.2
C16—C15—H15	120.5	C4—C5—H5A	111.2
C14—C15—H15	120.5	O1—C5—H5B	111.2
C15—C14—C13	121.18 (12)	C4—C5—H5B	111.2
C15—C14—H14	119.4	H5A—C5—H5B	109.1
C13—C14—H14	119.4	C7—C12—C11	121.95 (15)
C12—C7—C8	116.50 (12)	C7—C12—H12	119
C12—C7—C6	122.60 (11)	C11—C12—H12	119
C8—C7—C6	120.88 (12)	C10—C11—C12	119.98 (18)
C15—C16—C17	121.23 (12)	C10—C11—H11	120
C15—C16—Cl2	119.47 (10)	C12—C11—H11	120
C17—C16—Cl2	119.26 (11)	O2—C4—C5	104.37 (13)
O1—C2—O2	106.64 (11)	O2—C4—H4A	110.9
O1—C2—C6	112.75 (10)	C5—C4—H4A	110.9
O2—C2—C6	108.83 (11)	O2—C4—H4B	110.9
O1—C2—H2	109.5	C5—C4—H4B	110.9
O2—C2—H2	109.5	H4A—C4—H4B	108.9
C6—C2—H2	109.5	C11—C10—C9	119.91 (16)
C16—C17—C18	118.86 (12)	C11—C10—H10	120
C16—C17—H17	120.6	C9—C10—H10	120
C18—C17—H17	120.6	C2—O1—C5	104.87 (10)
C17—C18—C13	121.38 (12)	C2—O2—C4	108.24 (11)
			
C7—C6—C13—C18	−133.67 (12)	C14—C13—C18—C17	−1.9 (2)
C2—C6—C13—C18	96.78 (13)	C6—C13—C18—C17	177.41 (12)
C7—C6—C13—C14	45.63 (15)	C12—C7—C8—C9	−0.6 (2)
C2—C6—C13—C14	−83.91 (14)	C6—C7—C8—C9	−178.89 (13)
C16—C15—C14—C13	0.08 (19)	C12—C7—C8—Cl1	179.03 (10)
C18—C13—C14—C15	1.94 (18)	C6—C7—C8—Cl1	0.74 (17)
C6—C13—C14—C15	−177.38 (11)	C7—C8—C9—C10	0.6 (3)
C13—C6—C7—C12	−92.97 (14)	Cl1—C8—C9—C10	−179.00 (14)
C2—C6—C7—C12	35.70 (17)	C8—C7—C12—C11	−0.2 (2)
C13—C6—C7—C8	85.22 (14)	C6—C7—C12—C11	178.07 (13)
C2—C6—C7—C8	−146.12 (12)	C7—C12—C11—C10	0.9 (3)
C14—C15—C16—C17	−2.2 (2)	O1—C5—C4—O2	−28.32 (18)
C14—C15—C16—Cl2	175.65 (10)	C12—C11—C10—C9	−0.9 (3)
C7—C6—C2—O1	−74.72 (14)	C8—C9—C10—C11	0.1 (3)
C13—C6—C2—O1	53.83 (15)	O2—C2—O1—C5	−31.23 (15)
C7—C6—C2—O2	167.18 (10)	C6—C2—O1—C5	−150.62 (12)
C13—C6—C2—O2	−64.28 (13)	C4—C5—O1—C2	36.44 (17)
C15—C16—C17—C18	2.2 (2)	O1—C2—O2—C4	12.74 (16)
Cl2—C16—C17—C18	−175.63 (11)	C6—C2—O2—C4	134.64 (13)
C16—C17—C18—C13	−0.1 (2)	C5—C4—O2—C2	9.89 (18)

### Hydrogen-bond geometry (Å, º)

**Table:**

*D*—H···*A*	*D*—H	H···*A*	*D*···*A*	*D*—H···*A*
C6—H6···Cl1	0.98	2.57	3.0566 (13)	111
C12—H12···O1	0.93	2.38	3.046 (2)	128
